# Advantages of the Phosphatidylserine-Recognizing Peptide PSP1 for Molecular Imaging of Tumor Apoptosis Compared with Annexin V

**DOI:** 10.1371/journal.pone.0121171

**Published:** 2015-03-24

**Authors:** Soyoun Kim, Sang Mun Bae, Junyoung Seo, Kiweon Cha, Meilan Piao, Sun-Ji Kim, Hye-Nam Son, Rang-Woon Park, Byung-Heon Lee, In-San Kim

**Affiliations:** 1 Department of Biochemistry and Cell Biology, Cell and Matrix Research Institute, School of Medicine, Kyungpook National University, Daegu, Republic of Korea; 2 Division of high-risk pathogen research, Korea National Institute of Health, Korea Centers For Disease Control & Prevention (KCDC), Osong, Chungbuk, Republic of Korea; 3 Center for Theragnosis, Biomedical Research Institute, Korea Institute of Science and Technology, Seoul, Republic of Korea; University of Helsinki, FINLAND

## Abstract

A number of peptide-based indicators have been identified and reported as potential apoptosis probes, offering great promise for early assessment of therapeutic efficacy in several types of cancer. Direct comparison of the newly developed probes with previously used ones would be an important step in assessing possible applications. Here, we compared the newly identified peptide-based phosphatidylserine (PS) indicator PSP1 (CLSYYPSYC) with annexin V, a common probe for molecular imaging of apoptotic cells, with respect to PS binding kinetics, apoptotic cell-targeting ability, and the efficacy of homing to apoptotic tumor cells in a mouse model after treatment with the anticancer agent camptothecin. Our results indicate that PSP1 efficiently targeted apoptotic cells and generated apoptosis/tumor-specific signals after cancer treatment in the animal model, whereas a similar dose of annexin V showed weak signals. The formation of a stable complex of PSP1 with PS might be one reason for the efficient *in vivo* targeting. We suggest that PSP1 has potential advantages for *in vivo* apoptotic cell imaging and could serve as a platform for the development of *de novo* peptide-based probes for apoptosis.

## Introduction

Apoptotic cells generated in tissues are swiftly and safely removed by phagocytes under normal physiological conditions [1]. However, apoptotic cells are observed in tissues under pathological conditions such as cardiac diseases, including myocardial infarction, myocarditis, cardiomyopathy, cardiac allograft rejection, and atherosclerosis [2,3]. Apoptosis is also intentionally induced in cancerous or infected tissue as a therapeutic strategy. Clinical data indicate that radio- and chemotherapy cause rapid induction of apoptosis, peaking within 24 hrs of treatment [4–6]. Thus, *in vivo* imaging of apoptosis would have great value for assessing the therapeutic efficacy of cancer treatments as well as for diagnosing cardiac diseases at an early stage.

The exposure of phosphatidylserine (PS) on the outer surface of the plasma membrane bilayer, an early biochemical event in apoptosis, is an attractive target for molecular imaging of dying cells [7]. During chemo- and radiotherapy, subsequent necrosis also results in PS exposure due to disruption of the plasma membrane’s integrity [8]. One of the most successful and widely applied PS indicators is annexin V, an endogenous 36-kDa human protein that binds to PS with nanomolar affinity in a calcium-dependent manner [9,10]. The stability and biodistribution of annexin V derivatives depend on the radioisotope and the labeling modality used; several of these derivatives are undergoing clinical trials [11]. However, there are a number of limitations related to the application of annexin V in the clinical domain. First, because they are large protein drugs, annexin V derivatives have the potential drawbacks of poor stability during long-term storage; possible immunogenicity; and limited access to the target tissue, especially when the vasculature is disrupted by drug treatment [12]. The last aspect is particularly an issue in the monitoring of antiangiogenic therapy effects because many of these therapies are designed to disrupt cancer vascularization. Moreover, annexin V is not entirely specific for apoptotic cells. Annexin V targets necrotic as well as nonapoptotic cells with exposed anionic lipids under specific physiological or pathological conditions. Targeting necrotic cells may not be a critical disadvantage in the assessment of cancer treatment because a number of tumor-targeting agents cause necrosis and because all types of cancer therapy lead to a high degree of necrosis in the late stages. However, targeting nonapoptotic cells with low levels of exposed PS is a significant limitation, as this may lead to a low signal-to-background ratio [12].

The limitations of annexin V for apoptotic cell imaging have motivated a search for other molecular probes to be used in the clinical domain. Among them, small peptide probes targeting exposed PS generally have a lower affinity than protein probes do but tend to exhibit lower immunogenicity plus a more favorable biodistribution, in part because they more easily penetrate tumor tissue, even after disruption of the vasculature by tumor therapy. However, reports on peptide-based PS indicators in human and animal models are few, and direct comparisons of the efficacy of small-peptide PS indicators and annexin V in terms of their biochemical affinity and clinical relevance for cell death imaging have yet to be reported.

Using M13 phage display, a powerful approach for discovering small peptides [13–16], we successfully identified small peptides that specifically bind PS [17]. Here, we directly compared the peptide-based PS indicator PSP1 (CLSYYPSYC) with annexin V for molecular imaging of apoptotic cells. The PSP1 peptide specifically targeted apoptotic cells in tumors following systemic administration to camptothecin-treated tumor-bearing (H460 cell) mice in comparison with a similar dose of annexin V. Our results suggest that PSP1 offers benefits for molecular imaging of tumor apoptosis compared with annexin V and could be effectively developed into a *de novo* small molecular probe for imaging of apoptosis *in vivo*.

## Materials and Methods

### Materials

L-phosphatidylserine (PS, 840032), L-phosphatidylcholine (PC, 840055), and phytosphingosine (860499P) were purchased from Avanti Polar Lipids, Inc. Albaster, AL USA).

### Cell culture

The human lung cancer cell H460, which was obtained from the American Type Culture Collection (ATCC), was maintained in RPMI-1640 media supplemented with 10% heat-inactivated fetal bovine serum (FBS) and penicillin G/streptomycin at 37°C in a humidified atmosphere of 95% air and 5% CO_2_.

### Annexin V protein purification and fluorophore conjugation

Annexin V was cloned into the BamH1 and Xho1 sites of pET-28a (Novagen). His-tagged annexin V protein was expressed in an *Escherichia coli* strain (BL-21) and purified using Ni-NTA Agarose column chromatography (Qiagen, Inc., Valencia, CA, USA), as previously described [18]. Briefly, positive clones were cultured overnight at 37°C in LB medium (with 50 mg of kanamycin/ml), diluted 1:100, and grown until the optical density at 600 nm reached 0.5. At this point, 0.5 mM IPTG (isopropyl—D-thiogalactopyranoside) was added to induce expression of the fusion protein, and cells were incubated further for 4 hours. Expreesed annexin V was purified from the cell lysate using Ni-NTA Agarose column and subjected to SDS PAGE to examine the homogeneity. For fluorescence-activated cell sorting (FACS) and microscopic analyses, annexin V was coupled with fluorescein isothiocyanate (FITC; Sigma) following the manufacturer’s protocol. Briefly, the purified protein was incubated with FITC at a molar ratio of 1:1.5 at 37°C for 1 hr in a coupling buffer (50 mM sodium borate-NaOH (pH 9.0), 150 mM NaCl, and 1 mM EDTA). The labeling reaction was stopped by the addition of 50 mM glycine, and the mixture was dialyzed against 50 mM Tris-HCl (pH 8.0), 80 mM NaCl, and 1 mM EDTA to eliminate free FITC. For animal experiments, annexin V was conjugated to the near-infrared fluorescent dye Cy7.5 (GE Healthcare, Piscataway, NJ, USA).

Fluorescence-conjugated peptides were synthesized by the standard Fmoc method, conjugated with FITC at the N-terminus, and purified by HPLC (AnyGen Co., Gwangju, Korea). For *in vivo* experiments, after the peptides were synthesized, the fluorescent dye Cy7.5 was conjugated to the N-terminus of the peptides (AnyGen Co., Korea).

### Prediction of PSP1 peptide structure

The sequence of the PSP1 peptide was submitted to the PEP-FOLD server [19–21] for peptide folding, including PSIPRED prediction [22]. PEP-FOLD is a *de novo* approach aimed at predicting peptide structures from amino acid sequences.

### Flow cytometry analysis

To induce apoptosis of lung tumor cells (H460), the cells (5×10^6^ cell) were grown overnight and then treated with etoposide (50 μM) (Sigma) in serum-free media for 22 hrs, followed by washing with phosphate-buffered saline (PBS) and binding buffer (10 mM Hepes (pH 7.4), 150 mM NaCl, and 2 mM CaCl_2_). The apoptosis was validated using an annexin V-Alexa Fluor 488 kit (Invitrogen) and propidium iodide (PI) for FACS analysis. Normal or apoptotic cells (1×10^5^ cells/ml) were incubated with serial concentration (2, 10, 20, 50 μg/ml) of peptides or proteins in binding buffer (10 mM Hepes (pH 7.4) and 150 mM NaCl, with or without 2 mM CaCl_2_) at 4°C for 1 hr. The cells were washed with binding buffer for three times and immediately subjected to FACS analysis (FACScan, Becton Dickinson, San Jose, CA, USA).

### Confocal microscopy

H460 cells (2×10^4^ cells/well) were seeded and grown overnight on an eight-chamber slide (Nalgene Nunc Inc.) and then induced to undergo apoptosis with etoposide (50 μM), as described above. After washing with PBS, the cells were pre-incubated with 1% bovine serum albumin (BSA) containing binding buffer at 37°C for 30 min to block non-specific binding. The cells were incubated with 20 μg of fluorescein-labeled PSP1 peptide, annexin V, and control peptide at 4°C for 1 hr. The cells were counterstained with the nuclear stain 4',6-diamidino-2-phenylindole (DAPI) prior to mounting (Molecular Probes, Eugene, OR, USA) and examined using a laser confocal scanning system, consisting of a Leica TCS SP2 and a fluorescent microscope (Zeiss, Oberkochen, Germany).

### Surface plasmon resonance (SPR) analysis

To compare the kinetics of annexin V and PSP1 peptide binding to PS liposomes, SPR spectroscopy was employed (Reichert Inc, NY, USA). PC and PS liposomes (0.8:0.2 PC:PS) were generated as previously described [18] and were homogenously purified using a liposome mini-extruder with a 100 nm membrane filter, based on the manufacturer’s protocol (Avanti Polar Lipids). The PC and PS liposomes were captured on a carboxymethyl dextran chip (CMDH chip, 13206066) by a phytosphingosine layer, following the manufacturer’s suggestion. Briefly, phytosphingosine was immobilized by activating the carboxymethyl groups on dextran-coated chips (Reichert Life Sciences) through a reaction with N-hydroxysuccinimide (Sigma-Aldrich), followed by covalent bonding of the ligands to the chip surface via amide linkages and blocking of excess activated carboxyls with ethanolamine. The purified PS or PC liposomes were captured on the phytosphingosine layer at a flow rate of 5 μl/min in liposome immobilization buffer (10 mM Tris-HCl (pH 8.0), 100 mM NaCl, and 2 mM CaCl_2_). To use the PC-immobilized surface as a control, the injected PS or PC liposome volume was adjusted to an immobilization level of approximately 4000±200 RU. Different concentrations of the PSP1 peptide (0.313 to 5 μM) and annexin V (3.13 to 100 nM) in running buffer (10 mM Tris-HCl (pH 8.0), 100 mM NaCl, 2 mM CaCl_2_, and 62.5 μg/ml BSA) were allowed to flow over the surfaces bound to the immobilized liposomes for 3 min at a rate of 30 μl/min. The sensor surface was regenerated after each association and dissociation cycle by injecting 10 mM EDTA for 20 s, followed by 50 mM NaOH for 10 s. The interaction of the ligand and the analyte was analyzed using Scrubber 2 Biologic software (http://www.biologic.com.au/). The binding kinetics was assessed by determining the association (k_on_), dissociation (k_off_), and equilibrium (K_D_) constants.

### Animal model

All animal experiments were performed in compliance with institutional guidelines and according to the animal protocol approved based on the guidelines of the Institutional Animal Care and Use Committee (IACUC) of Kyungpook National University (permission No. KNU 2012-18). All efforts were made to minimize animal suffering.

Female nude mice (BALB/c nude; body weight, 20±3 g; n = 5) were housed in a specific pathogen-free environment at 22±2°C and 55±5% relative humidity with light. Tumors were generated by subcutaneously injecting H460 cells (5×10^6^ cells) into the right shoulder of 6-wk-old female BALB/c nude mice and allowing them to grow for 3 weeks (size of 0.5 to 0.7 cm). For *in vivo* analysis of tumor targeting, the tumor-bearing mice were either treated or not treated with a single dose of camptothecin (10 mg/kg) 24 hrs prior to intravenous injection of Cy7.5-labeled PSP1, Cy7.5-annexin V or Cy7.5-labeled control peptide into the tail vein under inhalational anesthesia (1% (w/v) isoflurane in 2 L oxygen).

*In vivo* fluorescence images were taken at different time intervals after peptide injection (10 min, 1 h, 3 h, 6 h, and 12 h) using an eXplore Optix system (ART Advanced Research Technologies Inc., Montreal, Canada), which has excitation/emission wavelengths of 778 nm/805 nm. The obtained images were processed using eXplore Optix OptiView Software and normalized based on the baseline fluorescence, assessed for each mouse prior to peptide injection.

### Tissue preparation

At the 6 hr time point, the animals were euthanized with CO_2_, and tumor tissues were removed from a subset of animals. The tumor tissues were then fixed by incubating with 4% paraformaldehyde (PFA) overnight, followed by freezing for cryosectioning and immunohistochemical studies under a fluorescence microscope (Zeiss, Oberkochen, Germany). Apoptosis in the tumor tissues was assessed by *in vitro* terminal deoxynucleotidyl transferase-mediated dUTP nick-end labelling (TUNEL) staining in accordance with the manufacturer’s instructions (Chemicon Int., Temecula, CA, USA).

## Results

### Structural model of PSP1 and FACS analysis of binding of FITC-labeled PSP1 and annexin V to apoptotic cells

**Fig 1:**
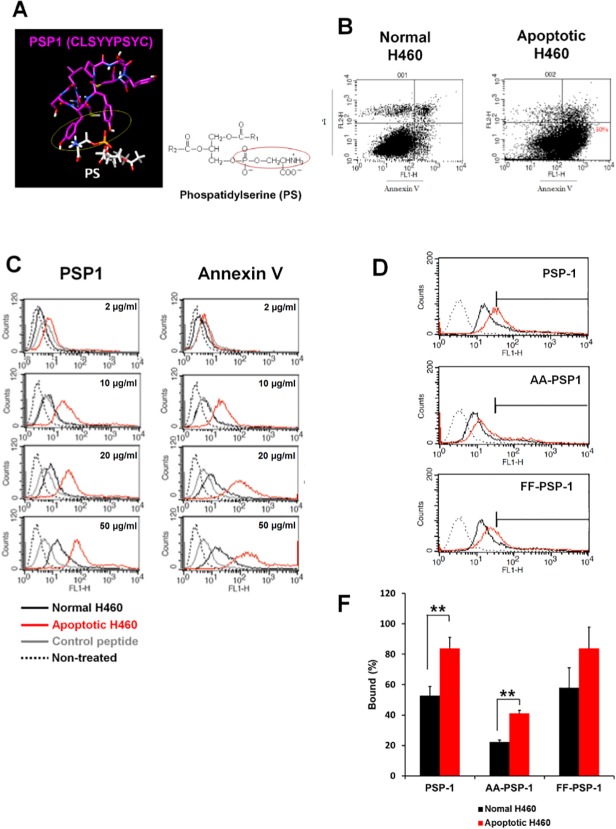
Structure of PSP1 peptide and binding of FITC-labeled PSP1 and annexin V to apoptotic cells. (A) PEP-FOLD simulations give the structure of the PSP1 peptide (magenta; hydroxyl group: red/white). PS (white) is displayed with its head group (phosphate: yellow; amide: blue) toward PSP1. (B) PS exposure on the cell membrane of apoptotic cells is examined by FACS analysis based on shifting following binding to annexin V-Alexa Fluor 488 (X-axis) and PI (Y-axis). (C) The indicated amount of FITC-CLSYYPSYC or FITC-annexin V was incubated with apoptotic H460 cells (red) or normal H460 cells (black) at 4°C for 1 hr. Control peptide (grey); FITC-NSSSVDK. (D) 10 μg/ml of FITC-PSP1 peptide (CLS**YY**PSYC) and mutants FITC-PSP1 (AA-PSP1; CLS**AA**PSYC. FF-PSP1; CLS**FF**PSYC) were incubated with apoptotic H460 cells (red) or normal H460 cells (black). (E) Bar chart indicates the FITC labeled peptide binding (under bar in D) to apoptotic H460 (red) vs. normal H460 cells (black). The results are presented as the means + s.d. (n = 3 independent examinations per group) **P<0.01.

The small peptide PSP1 was identified using the phage display technique on the basis of its specific interaction with PS on apoptotic cells [17]. By using the PEP-FOLD resource, the structure of the PSP1 peptide was investigated. This model suggested that the hydroxyl groups of two tyrosine residues in the middle of the sequence can participate in binding to the head group of PS (Fig. 1A). To assess the application of PSP1 as a molecular imaging probe, we directly compared it with the well-studied apoptotic cell marker annexin V. PSP1 was labeled with FITC at its N-terminus during peptide synthesis, and purified annexin V was conjugated to FITC, as described in the Materials and Methods section. The molar ratio of fluorescein to annexin V was determined to be 0.7. To compare the binding of PSP1 to apoptotic cells with that of annexin V, we first employed FACS analysis. Annexin V/PI staining showed that more than 50% of H460 cells underwent apoptosis in response to the addition of etoposide-containing media, without evidence of significant necrosis (Fig. 1B). In accord with previous studies, both PSP1 and annexin V specifically bound to apoptotic H460 cells in a concentration-dependent manner, in contrast to binding by control peptide (NSSSVDK) and binding to normal H460 cells (Fig. 1C). Annexin V appeared to exhibit a greater ability to bind to apoptotic cells, taking into account its molar ratio (the molecular weight of annexin V is 16-fold greater than that of PSP1) and labeling efficiency. To examine roles of tyrosine residues in the PSP1 peptide, two mutant PSP1 peptides (CLS**AA**PSYC and CLS**FF**PSYC) which consist alanine or phenylalanine instead of tyrosine were subjected to FACS analysis (Fig. 1D and 1E). The binding affinity of PSP1 peptide to apoptotic cells was significantly diminished by replacement of tyrosine by alanine. FF-substituted PSP1 bound to the apoptotic cells as much as PSP1 however it showed nonspecific binding to the normal cells, implying tyrosine residues play roles for binding to apoptotic cells.

### *In vitro* analysis of PSP1 and annexin V binding to PS liposomes using SPR

**Fig 2:**
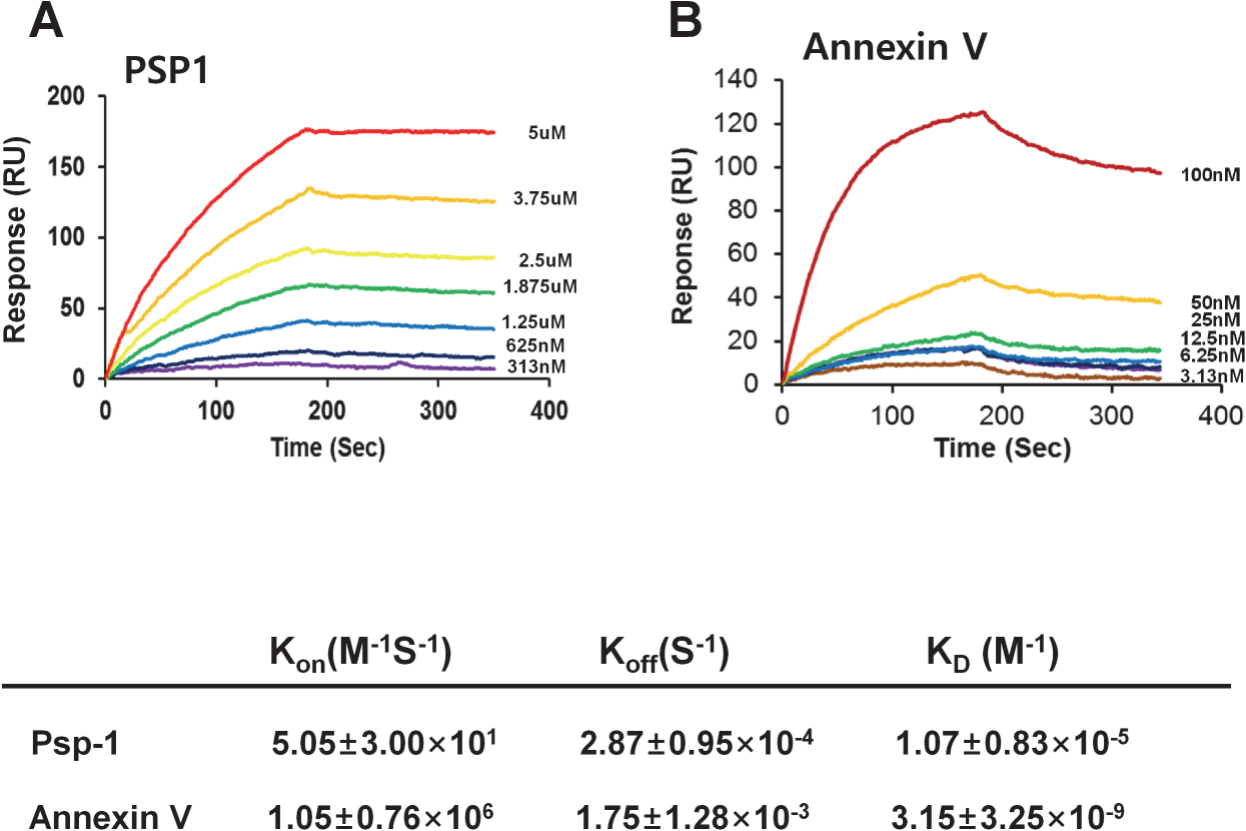
*In vitro* SPR analysis of PSP1 and annexin V binding to PS liposomes. (A) The indicated concentrations of PSP1 were injected onto the liposome-immobilized surface. Responses (RU) from the PC liposome-coated surface (reference) were subtracted from those from the PS:PC (2:8) liposome surface to monitor PS-specific binding. The equilibration binding constant (K_D_) and the association (K_on_) and dissociation (K_off_) rate constants were estimated using the Scrubber program. (B) Plots of the RU values of annexin V at the indicated concentrations.

The *in vitro* binding affinity of PSP1 and annexin V for PS was further examined using SPR analysis (Fig. 2). The SPR system does not just estimate equilibrium binding constants but also monitors binding events, or association (recognition) and dissociation (stability), in real time. We immobilized PS liposomes on the surface of an SPR chip, applied a series of different concentrations of PSP1 or annexin V, and examined the binding kinetics. PS-specific binding was determined by subtracting binding responses to a PC liposome-coated surface from those obtained using a PS liposome-coated reference surface. The *in vitro* binding affinity (equilibrium binding constant) of annexin V was 3.1 nM, whereas that of PSP1 was 10.7 μM, demonstrating that the biochemical affinity of annexin V was ∼3400-fold higher than that of PSP1. Although the affinity of PSP1 was significantly lower than that of annexin V, the real-time association and dissociation curves of PSP1 binding suggest that the peptide binds much more slowly to PS but, once bound, forms a more stable complex (slow dissociation) compared with annexin V.

### Microscopic analysis of FITC-labeled PSP1 and annexin V binding to apoptotic cells

**Fig 3:**
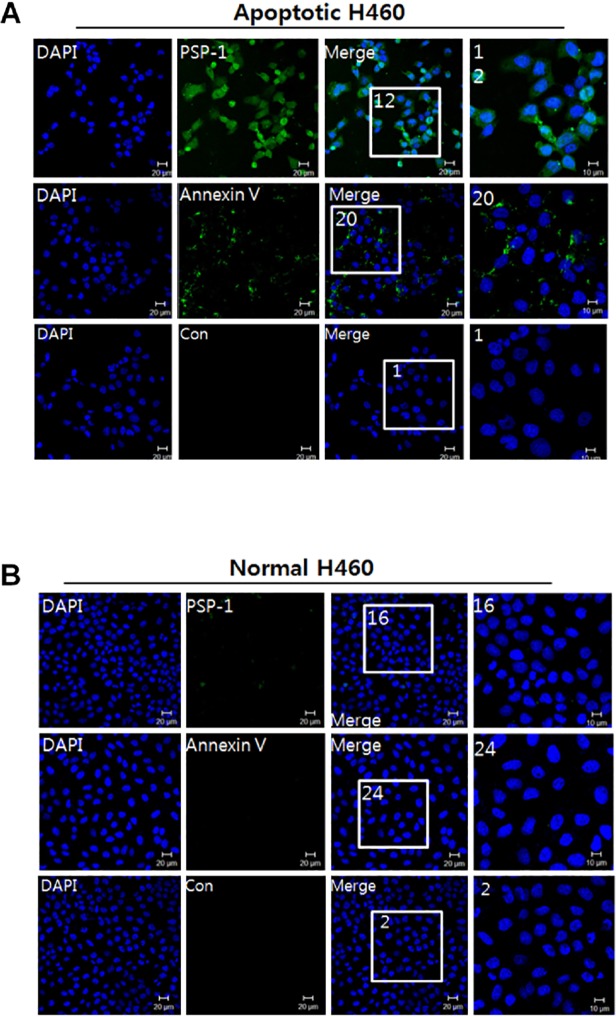
Microscopic analysis of binding of FITC-labeled PSP1 and annexin V to apoptotic cells. (A) Apoptotic H460 cells in a chamber slide were incubated with 20 μg of PSP1, control peptide, or annexin V for 30 min. The cells were washed and fixed with 4% PFA before staining with DAPI. The confocal images were taken by laser confocal microscopy. (B) Normal H460 cells were subjected to incubation with the same amount of peptide or annexin V.

Before comparing the *in vivo* binding of PSP1 and annexin V to apoptotic cells, we first examined their cell-binding capability *in vitro*. The specific targeting of PSP1 and annexin V to the cell membrane of apoptotic H460 cells was examined using confocal microscopy (Fig. 3). Under the conditions used, treatment of H460 lung cancer cells with etoposide caused PS exposure, which was examined by flow cytometry. This treatment did not significantly change the cell shape or number, indicating that these cells were undergoing early apoptosis. After incubation of apoptotic and normal H460 cells with PSP1 or annexin V at 4°C for 1 hr, apoptotic cell-specific signals were clearly detected in the PSP1-treated group, whereas signals in the annexin V-treated group were significantly weaker at the same concentration (20 μg/ml). These results were surprising because the 3400-fold higher binding affinity of annexin V would predict a 150-fold greater sensitivity at the same concentration, given a molar concentration 1/16^th^ that of PSP1 and the labeling efficiency of annexin V (0.7 FITC groups/molecule). This discrepancy can be partly explained by underestimation of the peptide’s binding affinity due to the low signal-to-noise ratio of peptide molecules in SPR analyses. However, these phenomena also suggest that the PSP1 peptide has benefits in PS-recognition applications *in vivo*, presumably through formation of a stable complex.

### *In vivo* imaging of tumors after induction of apoptosis

**Fig 4:**
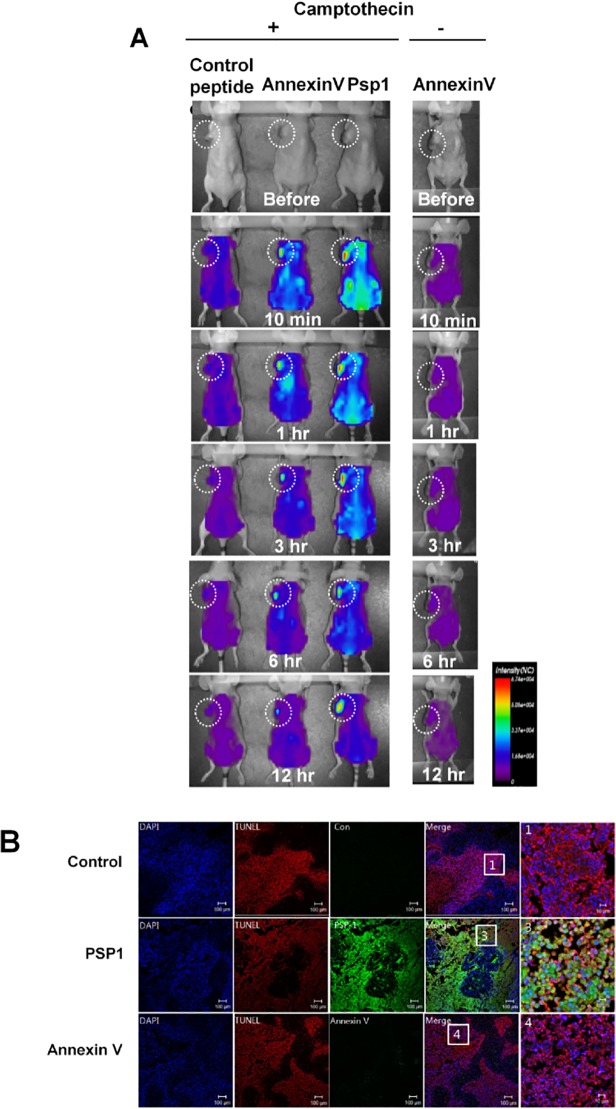
*In vivo* imaging of tumors after apoptosis induction. (A) Cy7.5-labelled PSP1peptide, control peptide, or annexin V was systematically injected into the tail vein of tumor-bearing nude mice treated with camptothecin 24 hrs before injection. The same amount of annexin V was also injected into non-camptothecin-treated tumor-bearing mice as a control. The homing of the peptides was examined after the indicated times using an optical imaging system. The white dotted circles indicate the tumors. (B) Histological examination of tumor tissues after homing of PSP1 and annexin V, performed under a fluorescence microscope. Tumor cell apoptosis was observed by TUNEL staining.

To test the apoptotic cell targeting of PSP1 and annexin V *in vivo*, we injected PSP1 or annexin V into tumor-bearing mice treated with the anticancer agent camptothecin, which acts primarily through induction of apoptosis. To better image probe homing *in vivo*, we labeled the PSP1 peptide, control peptide, and annexin V with a near-infrared fluorescent dye that has a longer wavelength and consequently deeper tissue penetration with lower background autofluorescence than FITC. On the basis of preliminary experiments, we selected probe concentrations of 100 μg/200 μl for annexin V and control peptide and 10 μg/200 μl for PSP1 to visualize the homing signals after equal image processing. Following intravenous injection of one of the labeled peptides or annexin V into tumor-bearing mice after chemotherapy, the *in vivo* fluorescence signals of annexin V as well as PSP1 were rapidly observed in the tumor region and sustained for more than 12 hrs (Fig. 4A). These signals seemed to be specific for apoptotic tumor cells because the same amount of annexin V did not home to the tumor region in the non-camptothecin-treated group. As for cellular targeting, the intensity of the tumor-homing signals of PSP1 appeared higher than those of annexin V for the entire measurement period. Given that the molar concentration of injected PSP1 (23.16 μM) was only 1.6-fold greater than that of annexin V (14.3 μM), these results indicate that PSP1 has advantages as a small peptide probe for apoptotic cell targeting in animal models. Systemically administered control peptide (100 μg/200 μl) did not show specific tumor-homing signals in camptothecin-treated tumor-bearing mice. Although the surrounding non-tumor tissues absorbed fluorescence at the early time points, this non-specific signal vanished over time.

### Histological examination of tumor tissues after *in vivo* PSP1 and annexin V homing experiments

After completion of the *in vivo* homing studies, cryosections were prepared from tumor tissues for histological examination. The tumor-homing fluorescence signal of the PSP1 peptide was significantly more prominent than that of annexin V, confirming that PSP1 targets apoptotic cells in tumor tissue with better efficacy than annexin V does (Fig. 4B). No signals were observed in tumor tissues from mice injected with control peptide. TUNEL staining confirmed an increased area of *in vivo* apoptosis in the tumors of camptothecin-treated mice.

## Discussion

During the past decade, considerable progress has been made in molecular imaging of apoptosis, and many novel imaging agents have been developed, offering great promise for early assessment of several types of cancer. One such agent is annexin V, and certain annexin V derivatives have progressed to clinical trials. However, the potential drawbacks of annexin V have prompted the development of small peptide-based imaging probes [23]. Direct comparison of imaging efficacy is an important step in evaluating the possible applications of these peptide probes in the clinical domain.

We previously identified PSP1 as a specific PS-binding peptide using phage display techniques and showed that it exhibits *in vivo* homing to apoptotic cells in tumor tissue. In the present study, we systematically compared the imaging ability of PSP1 with that of annexin V in terms of PS binding kinetics, apoptotic cell-targeting ability, and the efficacy of homing to apoptotic tumor cells after cancer therapy. The overall binding affinity (K_D_) of PSP1 was only 15 μM, which was significantly weaker than the nanomolar range of the binding affinity of annexin V. Interestingly, however, the dissociation of PSP1 was slow, even slower than that of annexin V, indicating that the peptide forms stable complex with PS, which is a notable characteristic as a peptide probe. *In vitro* FACS and SPR analyses showed that PSP1 specifically binds to apoptotic cells through PS exposed on the apoptotic cell surface. The AA- and FF-substituted PSP1 peptide analyses suggest possible mechanisms of this PSP1 binding; the two tyrosine residues play roles for targeting to apoptotic cells and the hydrophobic moieties may stabilize binding complex with cell membrane.

Microscopic analyses also showed specific targeting of PSP1 to apoptotic cancer cells, as was the case for annexin V. Although the affinity of annexin V for PS was significantly (∼10^3^-fold) greater than that of PSP1, our results demonstrated that the same concentration of PSP1 (20 μg/ml) targets apoptotic tumor cells more efficiently than annexin V does. We speculated that PSP1 forms a stable complex with PS and consequently tethers to apoptotic cells during incubation. The molecular binding mechanism of PSP1 has not yet been determined. No basic amino acids or putative Ca^2+^-binding amino acids are present in the PSP1 sequence, CLSYYPSYC, but two tyrosine residues in the middle might participate in binding to PS. Treatment with a higher concentration of PSP1 (40 μg/ml) resulted in a small degree of binding to untreated cancer cells (data not shown).

In animal experiments, PSP1 specifically homed to apoptotic cells in tumors after cancer therapy. The greater homing efficiency of PSP1 compared with annexin V was observed in tumor-bearing mice, suggesting that PSP1 has potential advantages for *in vivo* apoptotic cell imaging and could be developed as a *de novo* peptide probe for the detection of apoptosis. The formation of a stable complex of PSP1 with PS liposomes might be one reason for the dramatic *in vivo* targeting efficacy of PSP1.
